# Adaptation of a Mobile Interactive Obesity Treatment Approach for Early Severe Mental Illness: Protocol for a Mixed Methods Implementation and Pilot Randomized Controlled Trial

**DOI:** 10.2196/42114

**Published:** 2023-06-09

**Authors:** Ginger E Nicol, Madeline O Jansen, Amanda R Ricchio, Julia A Schweiger, Katie E Keenoy, J Philip Miller, Elaine H Morrato, Zhaohua Guo, Bradley A Evanoff, Joseph J Parks, John W Newcomer

**Affiliations:** 1 Department of Psychiatry Washington University School of Medicine Washington University in St. Louis Saint Louis, MO United States; 2 Division of Child & Adolescent Psychiatry Department of Psychiatry University of California, Los Angeles Los Angeles, CA United States; 3 Trial-CARE Division Center for Clinical Studies Washington University School of Medicine St. Louis, MO United States; 4 Division of Biostatistics Washington University School of Medicine Washington University in St. Louis St. Louis, MO United States; 5 Parkinson School of Health Sciences and Public Health Loyola University of Chicago Chicago, IL United States; 6 Division of General Medical Sciences Department of Internal Medicine Washington University School of Medicine St. Louis, MO United States; 7 National Council for Mental Wellbeing Washington DC, DC United States; 8 Thriving Mind South Florida Miami, FL United States

**Keywords:** community mental health services, implementation science, mental disorders, obesity, primary prevention

## Abstract

**Background:**

Obesity is common in individuals with severe mental illness (SMI), contributing to a significantly shortened lifespan when compared to the general population. Available weight loss treatments have attenuated efficacy in this population, underscoring the importance of prevention and early intervention.

**Objective:**

Here, we describe a type 1 hybrid study design for adapting and pilot-testing an existing mobile health intervention for obesity prevention in individuals with early SMI and Class I or early-stage obesity, defined as a BMI of 30-35.

**Methods:**

An existing, evidence-based interactive obesity treatment approach using low-cost, semiautomated SMS text messaging was selected for adaptation. Community mental health clinics and Clubhouse settings in Eastern Missouri and South Florida were identified to participate. This study has the following 3 aims. First, using the Enhanced Framework for Reporting Adaptations and Modifications to Evidence-based interventions, contextual aspects of the clinical and digital treatment environments are identified for adaptation, considering 5 main stakeholder groups (clinical administrators, prescribing clinicians, case managers, nurses, and patients). Following a 2-week trial of unadapted SMS text messaging, Innovation Corps methods are used to discover needed intervention adaptations by stakeholder group and clinical setting. Second, adaptations to digital functionality and intervention content will be made based on themes identified in aim 1, followed by rapid usability testing with key stakeholders. A process for iterative treatment adaptation will be developed for making unplanned modifications during the aim 3 implementation pilot study. Individuals working in partner community mental health clinics and Clubhouse settings will be trained in intervention delivery. Third, in a randomized pilot and feasibility trial, adults with 5 years or less of treatment for an SMI diagnosis will be randomized 2:1 to 6 months of an adapted interactive obesity treatment approach or to an attentional control condition, followed by a 3-month extension phase of SMS text messages only. Changes in weight, BMI, and behavioral outcomes, as well as implementation challenges, will be evaluated at 6 and 9 months.

**Results:**

Institutional review board approval for aims 1 and 2 was granted on August 12, 2018, with 72 focus group participants enrolled; institutional review board approval for aim 3 was granted on May 6, 2020. To date, 52 participants have been enrolled in the study protocol.

**Conclusions:**

In this type 1 hybrid study design, we apply an evidence-based treatment adaptation framework to plan, adapt, and feasibility test a mobile health intervention in real-world treatment settings. Resting at the intersection of community mental health treatment and physical health promotion, this study aims to advance the use of simple technology for obesity prevention in individuals with early-stage mental illness.

**Trial Registration:**

ClinicalTrials.gov NCT03980743; https://clinicaltrials.gov/ct2/show/NCT03980743

**International Registered Report Identifier (IRRID):**

DERR1-10.2196/42114

## Introduction

### Background and Rationale

Younger and treatment-naïve individuals with early severe mental illness (eSMI) experience adverse health effects associated with medications, including significant weight gain, metabolic dysregulation, and early onset cardiovascular disease [1,2]. eSMI patients are at high risk for treatment nonadherence, and benefit from behavioral programming that recognizes their unique engagement needs, which include psychological support to shore up healthy coping strategies and reduce cognitive load [3-5]. Successful health behavior change requires promotion of self-determination and cognitive control [6,7] as well as development of psychophysical self-knowledge through accurate interpretation of interoceptive cues (eg, hunger and satiety) [8,9]. These target mechanisms map onto the National Institute of Mental Health Research Domain Criteria [10] constructs for cognitive systems, cognitive control, and social processes (social awareness, self-awareness), offering a constructive pathway to achieve self-determination for health behaviors [11], cognitive control, and interoceptive awareness.

Digital or mobile health (mHealth) interventions show promise for enhancing engagement in lifestyle interventions among adolescents and young adults with chronic medical conditions [12-14]. Nonetheless, digital interventions to prevent or attenuate weight gain occurring early in the course of treatment remain understudied [15,16]. We have modified an existing interactive obesity treatment approach (iOTA) [17,18] involving collaborative goal setting with a health coach and interactive SMS text messaging to provide ongoing support and self-monitoring of behavior change goals in adults with severe mental illness (SMI) (iOTA-SMI) [19,20]. Adaptation of iOTA-SMI for a prevention focus in eSMI, however, requires unique target mechanism engagement, due to the cognitive and functional characteristics of patients with eSMI, who are still coming to terms with the brain system dysfunction caused by their illness.

**Figure 1:**
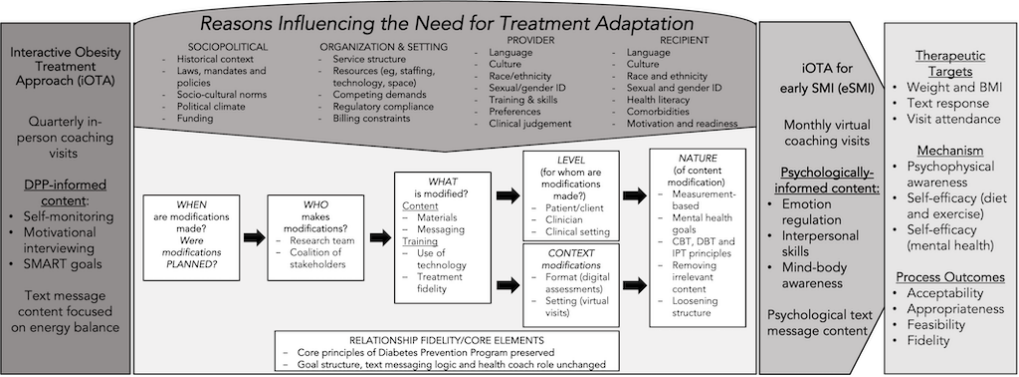
Framework for Reporting Adaptation and Modifications-Enhanced. CBT: Cognitive Behavior Therapy; DBT: Dialectic Behavior Therapy; DPP: Diabetes Prevention Program; eSMI: early severe mental illness; iOTA: interactive obesity treatment approach; IPT: Interpersonal Therapy.

Adaptation of effective interventions for new populations and settings (eg, eSMI and prevention) necessitates a formal evaluation process and implementation science framework. Stirman’s Framework for Reporting Adaptation and Modifications-Enhanced (FRAME, Figure 1) [21], offers an evidence-based approach for identifying adaptation needs based on contextual information that will inform adaptations specific to underrepresented racial, ethnic, socioeconomic and gender groups. Using FRAME, this study will further adapt the existing iOTA for obesity prevention in people with eSMI, hypothesizing treatment-related change in body weight will be inversely related to psychophysical awareness and self-efficacy for managing physical and mental health.

The mixed methods approach for this project involves the following three research aims to be conducted over 3 years: (1) identify adaptation targets to optimize iOTA for use by younger patients with eSMI treated in community mental health clinics (CMHCs) settings, incorporating perspectives from underrepresented groups; (2) adapt the existing iOTA, including modifications to key messaging to facilitate engagement and understanding in racial, ethnic socioeconomic, and gender-diverse groups in addition to adaptations relevant to eSMI in younger adults; and (3) conduct a randomized pilot test of the adapted iOTA in eSMI patients, evaluating both treatment effectiveness (change in body weight) and implementation effectiveness (feasibility, acceptability, and appropriateness) over 24 weeks.

### Trial Design

Employing the FRAME model, we will apply Innovation Corps (I-Corps) methods [22,23] to identify perceived barriers and challenges to implementing an iOTA in CMHC settings. Attention will be given to challenges related to the patient population (eg, early illness, cognitive impairment, and negative symptom effects on the ability to engage), care setting, case manager burden, lack of time and human resources, limited training, and supervision or accountability) and to the collection of multilevel (eg, patient, family, clinical staff, and administrator) and multimethod (eg, focus groups, semistructured interviews) data that can inform adaptations to increase the likelihood of success. Finally, key process outcomes will be measured (eg, acceptability, feasibility, fidelity, and appropriateness) during adaptation and pilot testing [24].

## Methods

### Aim 1: Identify iOTA Adaptation Needs for eSMI Using I-Corps Methods

This aim will determine how to best facilitate health behavior change in eSMI patients through interventions at the level of the individual, the health care provider, and the broader health care organization.

#### Study Settings

To achieve diversity in the participant samples, treatment settings, and social determinants, the proposed project will be conducted in CMHC and community Clubhouse settings in Missouri and Florida. Locations were chosen for the long-term collaboration between research teams and the representative racial and ethnic diversity of client and clinician populations and marked diversity of health care environments.

#### Study Procedures

##### I-Corps Methods

Starting with a list of hypotheses about barriers or challenges to implementation in CMHC settings, the study team identifies individuals who represent consumers or customers at multiple levels within the organization. Interviews and focus groups are conducted to test the hypotheses. Once facts have been obtained, the team reviews data looking for emergent themes that inform an overall “value proposition” for the customer or key stakeholder, identifying potential “pain” and “gain” areas. The team can then determine actions needed to change or adapt iOTA to best fit the CMHC environment. Purposive sampling methods will be applied to avoid bias in focus group responses, working with community partners to ensure group composition represents a broad range of perspectives from underrepresented populations.

##### Focus Groups

Once the focus group participants are identified, they will be asked to participate in one in-person or phone visit to set up health goals and related SMS text messages. They will then participate in 1-2 weeks of iOTA SMS text messaging to facilitate optimal feedback on intervention adaptations. Focus groups will be scheduled within 1 week of completing the 2-week iOTA trial. Groups will be conducted at the CMHC site during a time that is convenient for participants and will be facilitated by a research assistant facilitator and CMHC staff member taking additional notes.

##### Eligibility Criteria

We will conduct 8 focus groups of 5 participants each at each CMHC site, for a total of 16 focus groups (Table 1). Focus group composition will be monitored to balance groups with respect to age, race, and gender, taking care to include outlier perspectives on mHealth technology and obesity treatment. Participants will include administrators, prescribers, nursing staff, case workers, clients, and client social support.

**Table 1:** Focus group composition and questions.

Group type	Participant type	Areas of interest
Directors and Supervisors	Medical director, clinical supervisor	What should the qualifications and characteristics of the health coach be? Is it reasonable for the health coach to be an existing staff member?
Prescribing clinicians	Psychiatrists, advanced practice nurses	What type of training should health coaches receive? How often should face-to-face health coaching meetings be?
Nursing staff	Nursing staff, nurse care managers	What should the content of health coaching meetings be? How frequent should text messages be?
Community support workers	Community and peer support specialists	What should the content of the messages be? Is it helpful to have secure, 2-way texting capability?
Administrative staff	Supervisors and clinic managers	How much time could existing staff devote to intervention delivery? What would the optimal amount of time be for intervention effectiveness?
Members or clients	Adult clients (18-45 years of age)	What would make the intervention acceptable to younger adults? Are there modifications needed to address social determinants of health?

##### Data Management and Analytic Plan

Recorded groups will be transcribed, and text data will be entered into NVivo, coded by 2 independent raters. We will use inductive coding methods based on pragmatic-variant grounded theory to develop a coding tree based on the focus group question framework and new themes identified in the focus group transcripts. Precoding data exploration will identify the most used words and phrases by focus group, which are then explored for meaning and context to establish content themes.

### Aim 2: Adapt the Parent iOTA for Use in Younger Adults With eSMI

Contextual, content, and training aspects of the treatment will be adapted based on qualitative data collected using the I-Corps methods within the FRAME treatment adaptation model in aim 1. Adaptations will be tested for feasibility by members of the focus groups queried in aim 1.

#### Eligibility Criteria

Individuals who participated in aim 1 focus groups will be queried with individual semistructured interviews to obtain feedback on treatment adaptations.

#### Study Procedures

##### iOTA Adaptation

Existing libraries of SMS text messages will be tested for usability and cultural appropriateness among members of our target population of eSMI patients. We will conduct key informant interviews using the I-Corps methodology following this testing and adapt the messages and risk assessment platform as needed based on this information.

##### Key Informant Interviews

Using a standardized interview guide developed using I-Corps methods, we will conduct semistructured, in-person key informant interviews with 1-2 members from each focus group at each study site to obtain impressions about adaptations made based on focus group results. Interviewing will be curtailed when saturation occurs (ie, when the same themes repeatedly emerge) [25,26].

##### Data Management and Analytic Plan

Interviews will be digitally recorded and transcribed verbatim. Data will be analyzed using standard content analysis strategies listed in aim 1.

### Aim 3: Randomized Controlled Pilot Test of the Adapted Intervention

**Figure 2:**
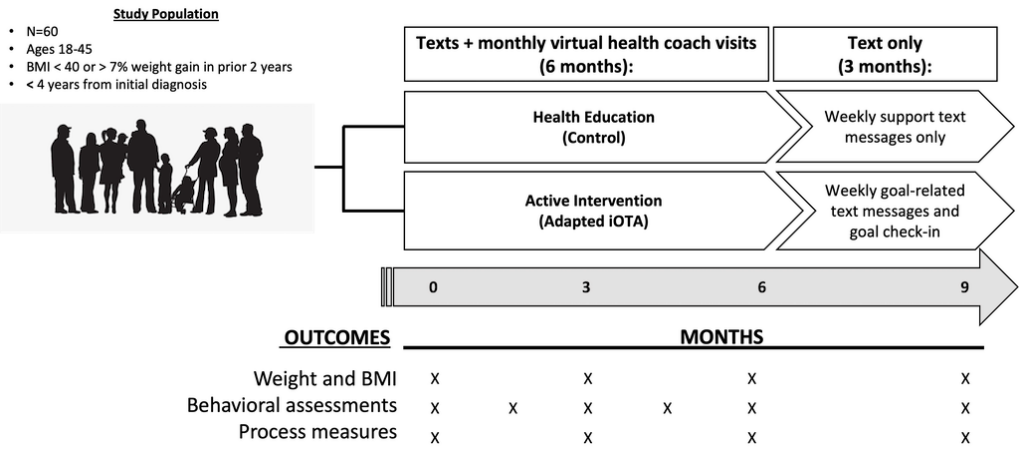
Study overview. iOTA: interactive obesity treatment approach.

In this aim, we will conduct a randomized 24-week pilot study of the adapted iOTA-eSMI intervention, followed by 12 weeks of SMS text messaging intervention alone in 60 participants (30 at each study site), evaluating both intervention and implementation effectiveness (Figure 2).

#### Eligibility Criteria

To capture “early” phase mental illness, we will use the length of illness requirement commonly used in Food and Drug Administration Phase III clinical trials testing treatment efficacy in early mental illness (eg, less than 5 years from diagnosis). “At risk” weight is categorized using the Food and Drug Administration definition of clinically significant weight gain (eg, more than 7%) or overweight or early Class 1 obesity by BMI.

#### Study Procedures

##### Screening Assessments

To ensure minimal cognitive capacity to understand treatment materials and engage in SMS text messaging, we will use the Rapid Estimate of Adult Literacy in Medicine-Short Form (REALM-SF) minimal score of 5 (consistent with 7th grade reading level) and the University of California San Diego Brief Assessment of Capacity to Consent score of 15 or higher (consistent with the capacity to consent). To ensure minimal psychiatric symptom stability, we will use the Clinical Global Impression-Severity scale minimal score of 4 (consistent with moderate illness severity) or lower. The schedule of study visits and assessments is presented in Table 2.

**Table 2:** Schedule of assessments.

		Enrollment	Allocation	Postallocation								Closeout
		Screen and startup	Baseline	T1	T2	T3	T4	T5	T6	T7	T8	
Study week		−2	0	1	4	8	12	16	20	24	36	
**Screening and baseline assessments**												
	Informed consent^a^	✓										
	CEI^b^	✓										
	CGI^c^	✓									✓	
	Medication reconciliation	✓	*^d^	*	*	*	*	*	*	*	✓	
	Health status questionnaire	✓									✓	
	Height, weight, BMI	✓										
	PASS^e^		✓								✓	
	Self-efficacy^f^		✓								✓	
	1-week Fitbit activity	✓	✓								✓	
	Treatment allocation		✓									
	Time spent (hours)	1	1.5	0.15	0.15	0.15	0.15	0.15	0.15	0.15	1	
**Study visit assessments**												
	Adherence (visit attendance)	✓	✓	✓	✓	✓	✓	✓	✓	✓	✓	
	Height, weight and BMI	✓	✓	✓	✓	✓	✓	✓	✓	✓	✓	
	Feasibility and acceptability							✓			✓	
	Treatment satisfaction							✓			✓	
	Time spent (hours)	0.15	0.15	0.15	0.15	0.15	0.15	0.15	0.15	0.15	1.5	
**mHealth assessments**												
	Adherence (% SMS text message response)			✓	✓	✓	✓	✓	✓	✓	✓	
	Weekly body weight (lbs)			✓	✓	✓	✓	✓	✓	✓	✓	
	Weekly goal progress			✓	✓	✓	✓	✓	✓	✓	✓	
	Time spent (hours)	0	0	0.15	0.15	0.15	0.15	0.15	0.15	0.15	0.15	
**Health coach self-assessments**												
	Response to intervention			✓	✓	✓	✓	✓	✓	✓	✓	
	Treatment fidelity			✓	✓	✓	✓	✓	✓	✓	✓	
	Time spent (hours)	0	0	0.15	0.15	0.15	0.15	0.15	0.15	0.15	0.15	

^a^Ability to provide Informed Consent assessed via the Rapid Evaluation of Adult Literacy in Medicine-Short Form and University of California, San Diego Brief Assessment of Capacity to Consent.

^b^CEI: credibility and expectations for improvement.

^c^CGI: clinical global impression scale.

^d^Assessments done as needed based on clinician judgment.

^e^PASS: psychophysical awareness and skills scale.

^f^Self-efficacy: self-efficacy for mental health, diet and physical activity.

##### Primary and Secondary Outcomes

The overall goals of this study are to establish the feasibility and acceptability of iOTA-eSMI as well as to inform sample size calculations for future randomized studies. Thus, the primary outcome will be a change in body weight (lbs) during 24 weeks of randomized treatment. Secondary outcomes will include changes in BMI, psychophysical awareness and skills, and self-efficacy for mental and physical health [27-29]. An increase in self-efficacy and psychophysical awareness are the proposed mechanisms of action for active iOTA intervention.

##### Psychophysical Awareness and Skills Scale

This composite measure draws from research domain criteria domains of cognitive systems and social processes, including cognitive control, perception, and understanding of self, self-awareness, self-monitoring, and self-knowledge. The composite measure includes 10 items that assess cognitive control [30], self-determination [31,32], and interoceptive awareness [33].

##### Self-Efficacy for Eating and Exercise Habits

For this study, we will use abbreviated surveys developed by Sallis et al [11] to measure self-efficacy in health behaviors involving eating and physical activity. Self-efficacy for diet and exercise will be measured at each study visit.

##### Mental Health Self-Efficacy Scale

The Mental Health Self-Efficacy Scale assesses belief in one’s capability to perform behaviors related to mental health self-care and has been shown to predict response to mHealth interventions for mental health [27]. The Mental Health Self-Efficacy Scale will be completed at each in-person health coaching visit [28].

##### Exploratory Outcomes

We will characterize intervention feasibility, engagement, and implementation challenges as measured by enrollment, retention, and visit adherence; intervention acceptability [5,34]; engagement as measured by visit attendance and SMS text messaging response rates; health coach self-assessment of competence and fidelity to the intervention [5,35]; and intervention appropriateness and acceptability [36,37] as rated by clients and staff. Finally, participants will be provided a Fitbit Flex to be worn for 1 week before study visits 1 (baseline) and 7 (24 weeks) to assess for change in physical activity through step counts. Data will be downloaded upon return of the device.

### Study Interventions

#### Parent iOTA

The individual-level iOTA intervention for adults with an SMI [20,38] is based on assessment of individual behavior risks, collaborative goal setting with a health coach, and use of an interactive SMS text messaging system that prompts participants to report their weight and their progress on achieving their goals with a “check-in” day each week, and sends immediate, tailored feedback about their progress. Weekly and monthly health tip messages are customized to the participant’s selected health goals. If a participant is making progress on their goal, the SMS text messaging system provides an opportunity to increase goal difficulty between health coaching sessions.

#### iOTA-eSMI Active Intervention

Monthly in-person visits consist of assessing current health behaviors, working collaboratively with the client to identify desired behavior changes, then using a list of existing goals with a stepped level of difficulty based on the participant’s current behaviors, the client and health coach collaboratively select up to 3 goals for the next month. These goals have prescribed SMS text messaging tailored to the level of difficulty to support the participant daily with health behavior change [20].

#### Health Education

The Health Education attentional control condition matches iOTA in time and number of sessions. Participants randomized to the control conditions will receive monthly in-person health coaching visits and a weekly SMS general “Health Tip” text message. Visits will be structured according to the current US Preventive Services Task Force recommendations to counsel participants on energy balance, physical activity, and nutrition [39,40], but will not involve formal goal setting.

### Ensuring Rigor and Reproducibility

#### Treatment Fidelity

Health coach adherence to the intervention protocol is determined by structured, direct observation of individuals carrying out the behavioral intervention, evaluating for evidence of (1) adherence to the treatment protocol and (2) competence in the treatment delivery. Health coaches will self-monitor treatment fidelity as noted above, and implementation challenges will be discussed during weekly study teleconferences.

#### Outcomes

Both clinical and implementation-related outcomes will be measured, with the primary endpoint being 24 weeks. Clinical outcomes of interest are related to obesity either directly (weight, BMI), or indirectly (self-efficacy, psychophysical awareness). Implementation outcomes include engagement, acceptability, appropriateness, and fidelity.

#### Recruitment

Targeted educational and recruitment in-services will be conducted at clinical sites in Missouri and Florida. The goal of the in-service will be to make clinicians and clinical administrators aware of this project and to assist in identifying eligible participants. Recruitment and retention tracking across sites will be reviewed on weekly study team conference calls between the sites to identify recruitment shortfalls and broaden recruitment efforts.

#### Randomization Strategy

As it is anticipated that participants in the health education group will either continue to gain weight or at least maintain their current weight, participants will be randomized 2:1 to either the iOTA-eSMI intervention or to the health education control condition. Groups will be balanced to reflect the age, gender, racial, and ethnic distribution of the client base at each site.

### Data Collection, Management, and Analysis

#### Data Collection Methods

All data will be entered into the Research Electronic Database Capture (REDCap) database through a secure web portal and stored on secure servers. Databases are built with multiple layers of security, following best practices and institutional requirements for securing sensitive data. All automatic system-generated messages in the iOTA program will be sent directly to participants’ mobile phones through a web-based app developed and supported by a Health Insurance Portability and Accountability Act–compliant digital platform that has been approved by the institutional review board (IRB) [19].

#### Planned Primary and Secondary Analyses

The primary outcome for this study will be mean change in body weight (lbs) after 24 weeks of iOTA-eSMI or Health Education. Secondary outcomes include a change in BMI, psychophysical awareness and skills, and health-related self-efficacy. We will use a repeated measure mixed model to look at trajectory over the study course with a time × treatment interaction estimating treatment effect. Structural equation growth models will be used to estimate the correlation between the trajectory of psychophysical skills and related self-efficacy and that of weight or BMI.

#### Exploratory Analyses

Additional analyses will focus on characterizing feasibility, engagement, and implementation challenges as measured by enrollment and retention; visit adherence and text response rates; client expectations for improvement [41]; fidelity of treatment delivery; client and staff-rated feasibility, appropriateness, and acceptability. Exploratory analyses will also evaluate the relationship between mental health self-efficacy and psychotropic medication compliance and iOTA treatment compliance or engagement.

### Monitoring

#### Data Monitoring

All electronic files (eg, database, spreadsheet, etc) containing identifiable participant information are password protected. Databases that contain private health and identifiable information are behind firewalls and require a password or username for access. All data will be entered into the REDCap [42] through a secure web portal. Databases are built with multiple layers of security, following best practice requirements for securing sensitive data. Conditional mean imputation will be used to account for missing data.

#### Harms

##### Potential Risks

All participants will be informed of potential risks before enrollment. Participation is voluntary, and the participant may choose not to participate in the research study or to withdraw at any time.

##### Prevention of Risk

All data collected from participants participating in the intervention will only be used for research purposes. Only members of the research team will have access to the data collected as part of this proposed study. No individual’s data will be shared outside of the research team. Participants will be advised that their participation in the research study is completely voluntary, that they may choose not to participate in the study, and that they may withdraw their participation in the research study at any time without impacting their ongoing psychiatric or medical care.

#### Auditing

Before study enrollment, all current and ongoing health issues and medications will be identified and documented. Over the course of the study, participants will be asked to report any health events, changes in medication, and hospitalizations, and a determination will be made as to whether these may be related to their participation in the study protocol and/or intervention. Follow-up surveys will contain questions on potential adverse events (AEs), and interactions with the health coach will include specific inquiries about potential AEs. We will track the dates of each event, intervention needed (if applicable), severity, and the likelihood that the event is related to the study.

#### AE Definitions

An AE is defined as any untoward medical occurrence in a participant that is temporally associated with participation in the clinical study, whether it is attributed to the study. Unanticipated AEs are those occurrences not noted in the study consent form. A severe adverse event (SAE) will be defined as an AE that is life-threatening, requires inpatient hospitalization, and/or results in death or severe disability.

#### AE Reporting

All AEs and SAEs will be reviewed within 72 and 24 hours, respectively. Reports will be generated for each event and will include a description of the event, when and how it was reported, as well as any official chart records or documentation to corroborate the event, and determination of attribution. AE and SAE reports will then be sent to the IRB and the National Institutes of Health (NIH) program official.

#### Reporting Timeframes

Any “related” or “possibly related” deaths will be reported within 1 day of when we become aware of the event. Unanticipated AEs will be reported to the IRB within 10 working days. Any study-related SAE will be reported to the NIH within 2 weeks; all others will be included in the annual report to NIH. Anticipated AEs will be reported to the IRB at the time of continuing review. Any action resulting in temporary or permanent suspension of study conduct will be immediately reported to the appropriate NIH program official.

#### Safety and Study Progress Reviews

Oversight of data collection, management and intervention development, and delivery will be provided during weekly supervisory teleconferences with the study team delivering the intervention during the pilot study.

### Ethics Approval

#### Research Ethics Approval

The Washington University Institutional Review Board (IRB) approved the focus groups (IRB ID: 201907136) and the randomized pilot study (IRB ID: 201911123).

#### Protocol Amendments

Protocol versions and related changes are listed in Table 3.

**Table 3:** Protocol version, date, and description of amendment content.

Version	Approval date	Protocol modifications
1.0	December 9, 2019	Initial protocol approval
1.1	December 16, 2019	Updated recruitment materials, modification of total planned enrollment
1.2	May 6, 2020	Added and removed research staff; added a clinical site in Missouri; added option for doing “in-person” study visits virtually or by phone; added verbal consent by phone and waiver of written consent if no access to a computer; rationale for no power calculation (pilot and feasibility study) added to the analysis plan
1.3	June 5, 2020	Updated consent language
1.4	December 16, 2020	Added and removed research staff; updated inclusion and exclusion criteria–increased upper age limit to 60 years from 45 years; removed the REALM-SF^a^ from consent process; removed exclusion for stable or well-managed type 2 diabetes; added recruitment from EPIC
1.5	March 17, 2021	Added research staff
1.6	November 5, 2021	Added and removed research staff; added a clinical site in Florida
1.7	November 27, 2021	Added research staff
1.8	March 4, 2022	Added research staff; added methods for sending test and secure, encrypted emails as well as option for participants to opt out of encrypted email communication with study staff (eg, can authorize emails to be sent without encryption)
1.9	June 9, 2022	Added research staff

^a^REALM-SF: Rapid Estimate of Adult Literacy in Medicine-Short Form.

#### Consent and Assent

Each participant will be administered the REALM-SF to estimate literacy and the University of California, San Diego Brief Assessment of Capacity to Consent to establish understanding of the study and active treatment. Prior to randomization, all participants will undergo 1 week of SMS text messaging to ensure that they are able to receive and appropriately respond to SMS text messages.

#### Confidentiality

The potential risk to privacy and/or confidentiality is that protected health information may be accidentally disclosed outside of the study. To avoid sending messages to the wrong number, the research team will first send a test message to ensure that messages are being sent to correct number. Participants will also be encouraged to use passcode protection to keep information on their phones private.

#### Access to Data

All data collected as part of the study will be coded with a unique study identification number. Only research study staff trained in the Health Insurance Portability and Accountability Act, Collaborative Institutional Training Initiative, and Good Clinical Practice training will have access to the data collected. At each site, data collected for this study will be stored in locked files in locked offices and only designated members of the research team will have access to the files.

#### Ancillary and Posttrial Care

Participants will continue to receive primary medical and psychiatric care as usual from their existing treatment teams during study participation, and health coaches will communicate any relevant information on their medical and psychiatric progress to treatment teams as authorized by the participant to coordinate care. At the conclusion of the study, participants will remain in the care of their existing treatment teams.

#### Dissemination Policy

The sharing of information created by this project will follow the NIH’s requirement for sharing raw, descriptive, and analyzed data (positive and negative) with the National Institute of Mental Health (NIMH) Data Archive via the National Database for Clinical Trials Related to Mental Illness. The data generated by NIH funding and other support will be presented at national or international scientific conferences and published in peer-reviewed journals. All publications will abide by the NIH Public Access policy, and any unique research resources or tools resulting from the project will be made readily available for research purposes to qualified individuals within the scientific community after publication and upon request.

## Results

The characteristics of focus group participants from aim 1 are outlined in Table 4. The Community Support Specialist group included Peer Specialists (6/16, 38% of the overall support specialist group). The “Prescriber” group included psychiatrists (7/9, 78% of the prescriber group) and psychiatric nurse practitioners (2/9, 22%).

At the time of publication, a total of 52 participants have been enrolled in aim 3. The baseline characteristics are reported in Table 5.

**Table 4:** Baseline participant.

Characteristic		Participants (N=72)
**Participant demographics**		
	Located in Eastern Missouri, n (%)	45 (62.5)
	Located in South Florida, n (%)	27 (37.5)
	Age (years), mean (SD)	41.9 (12.0)
	Male, n (%)	22 (30.6)
	Female, n (%)	46 (63.9)
	Preferred not to answer (sex), n (%)	4 (5.6)
	White, non-Hispanic, n (%)	30 (41.7)
	White, Hispanic, n (%)	9 (12.5)
	Black, non-Hispanic, n (%)	22 (30.6)
	Asian, non-Hispanic, n (%)	2 (2.8)
	American Indian or Alaskan Native, n (%)	1 (1.4)
	Preferred not to answer (race), n (%)	3 (4.2)
	More than 1 Race, non-Hispanic, n (%)	1 (1.4)
	More than 1 Race, Hispanic or Latino, n (%)	3 (4.2)
**Clinic role, n (%)**		
	Clinical supervisor or director	26 (36.1)
	Prescribing clinicians	9 (12.5)
	Community support specialist	16 (22.2)
	Administrative staff	8 (11.1)
	Nursing Staff	1 (1.4)
	Client or member	12 (16.7)

**Table 5:** Baseline participant characteristics, aim 3.

Variable			Total (N=52)		iOTA^a^ (n=33)		Education (n=17)
**Demographics**							
	Age (years), mean (SD)	38.2 (11.2)		39.8 (11.3)		37.4 (11.2)	
	Male, n (%)	21 (42)		16 (48)		5 (29)	
	Female, n (%)	29 (58)		17 (52)		12 (71)	
	Caucasian (White), n (%)	17 (34)		12 (36)		5 (29)	
	African American (Black), n (%)	24 (48)		15 (45)		9 (53)	
	Other (Mexican American), n (%)	1 (2)		1 (3)		0 (0)	
	Other (multirace), n (%)	2 (4)		1 (3)		1 (6)	
	Other (not specified), n (%)	2 (10)		2 (6)		0 (0)	
	Prefer not to answer (race), n (%)	4 (8)		2 (6)		2 (12)	
	Hispanic, n (%)	12 (24)		9 (27)		3 (18)	
	Non-Hispanic, n (%)	33 (66)		21 (64)		12 (71)	
	Prefer not to answer (ethnicity), n (%)	5 (10)		3 (9)		2 (12)	
**Primary diagnosis, n (%)**							
	Schizophrenia spectrum disorder	25 (52)		18 (55)		7 (41)	
	Bipolar disorder	16 (33)		11 (33)		5 (29)	
	Major depressive disorder	14 (29)		7 (21)		7 (41)	
	Anxiety disorder	8 (16)		3 (9)		5 (29)	
	Posttraumatic stress disorder	7 (15)		4 (12)		3 (18)	
	Autism spectrum disorder	2 (4)		2 (6)		0 (0)	
**Baseline values of key outcome measures, mean (SD)**							
	Weight (lbs)	254.5 (75.4)		252.4 (75.8)		257.7 (77)	
	BMI	40.8 (11.8)		39.8 (11.7)		42.3 (12.2)	
	Clinical global impression severity scale	3.7 (0.7)		3.7 (0.6)		3.6 (0.8)	
	Self-efficacy for diet and exercise	120.2 (28.6)		116.5 (31.3)		129.0 (19.3)	
	Self-efficacy for mental health	40.4 (13.1)		41.3 (13)		38.7 (13.9)	
	Psychophysical awareness and skills scale	34.5 (4.8)		34.2 (4)		34.9 (6)	
**Device type, n (%)**							
	Smartphone	48 (94)		32 (97)		16 (94)	
	Cell phone with SMS text messaging capability	3 (6)		1 (3)		1 (6)	
**Health insurance, n (%)**							
	Medicaid	31 (62)		22 (67)		9 (53)	
	Medicare	6 (12)		3 (9)		3 (18)	
	Commercial	8 (15)		3 (9)		4 (24)	
	Other (both Medicaid and Medicare)	1 (2)		1 (3)		0 (0)	
	Prefer not to answer	4 (8)		3 (9)		1 (6)	

^a^iOTA: interactive obesity treatment approach.

## Discussion

Early-onset obesity and premature mortality due to cardiovascular disease are more prevalent in mentally ill persons, who experience many barriers to engaging in healthy lifestyle behaviors. Furthermore, communities of color, and those who live with chronic medical conditions like SMI and obesity are disproportionately affected, and particularly those living in underserved areas, may not have access to high-quality health promotion programs. Here, we describe the design of a hybrid pilot and feasibility study evaluating both the effects of a mHealth obesity prevention program on body weight and the implementation of an intervention in real-world clinical settings.

The study design and baseline characteristics of the enrolled participant population for aims 1 and 3 largely reflect what has been reported in other studies of individuals with SMI. Participants who reported ethnicity as Hispanic or Latino (12/72, 17%) is similar to the 19% reporting any mental illness in the 2020 Census Bureau but lower than the number of individuals of Hispanic or Latino ethnicity reporting any mental health disorder in 2020 (27%) [43]. The proportion of focus group participants who reported race as Black or African American (22/72, 31%) was higher than in the overall US population (12%) [44] but is similar to the proportion of Black Americans reporting any mental health disorder (28%) in the 2020 National Survey on Drug Use and Health [43]. The proportion of individuals enrolled and randomized in aim 3 who were assigned as female at birth (29/52, 58%) and those of Hispanic ancestry (24/52, 24%) are consistent with what has been reported in large population-based studies [43,45,46]. However, the proportion of African American participants represented in the overall study sample (24/52, 48%) is higher than what has been reported in larger studies of both the prevalence of SMI [43,47] and the prevalence of obesity in the general population [48]. Finally, the mean age of 38.2 (SD 11.2) years and BMI of 40.8 (SD 11.8) at baseline were both on the higher end of the range allowable by inclusion criteria.

This study is subject to important limitations. First, although not completed, the sample size for the study is small, and therefore, subgroup analyses are unlikely to yield interpretable results. Second, the results may not be generally applicable in all contexts. That the enrolled population in this study is predominantly Black and, in general, physically and psychiatrically more symptomatic at baseline than expected could be explained in part by timing. Enrollment began at the height of the COVID-19 pandemic when community clinics across the country were shut down to new admissions and in-person visits [49]. Thus, the higher than expected physical and psychiatric comorbidities at baseline, a greater proportion of Black participants, and higher mean age than expected likely reflect the now well-described disproportionate impact the pandemic had on individuals with SMI from underrepresented communities [49].

Obesity prevention programs that use technology as the primary intervention delivery method may increase patient engagement with health promotion programs in general, in addition to reducing care delivery costs and increasing accessibility. This is especially relevant in the era of COVID-19 infection control precautions, which favor remote health care delivery. By pilot-testing this intervention in real-world clinical settings, we aim to adapt the intervention in real-time based on the prominent challenges and barriers that arise in the CMHC context. The results of this study will inform not only how such interventions may need to be modified for optimal effectiveness in eSMI, but also help us to understand how digital and mHealth interventions can be adapted for use in the unique ecosystems of clinical care delivery in community mental health settings, where implementation of digital interventions has been limited.

## Abbreviations

AE: adverse event
CMHC: community mental health clinics
eSMI: early severe mental illness
FRAME: Framework for Reporting Adaptation and Modifications-Enhanced
I-Corps: Innovation Corps
iOTA: interactive obesity treatment approach
IRB: institutional review board
mHealth: mobile health
NIH: National Institutes of Health
NIMH: National Institute of Mental Health
REALM-SF: Rapid Estimate of Adult Literacy in Medicine-Short Form
REDCap: research electronic database capture
SAE: severe adverse event
SMI: severe mental illness

## Data Availability

Dissemination (ie, sharing) of all data created by this study is an explicit goal of the overall project. Thus, raw, descriptive, and analyzed data (positive and negative) will be uploaded to the National Institute of Mental Health Data Archive via the National Database for Clinical Trials Related to Mental Illness. This process is part of our data safety and management plan for the study. All publications will abide by the National Institutes of Health Public Access policy, and any unique research resources and tools will be made readily available for research purposes to qualified individuals within the scientific community after publication and upon request.
